# Influence of local epidemiology on the performance of common colistin drug susceptibility testing methods

**DOI:** 10.1371/journal.pone.0217468

**Published:** 2019-06-06

**Authors:** Lucia Asar, Susanne Pfefferle, Marc Lütgehetmann, Armin Hoffmann, Juri Katchanov, Martin Aepfelbacher, Holger Rohde, Florian P. Maurer

**Affiliations:** 1 Institute of Medical Microbiology, Virology and Hygiene, University Medical Center Hamburg-Eppendorf, Hamburg, Germany; 2 Department of Intensive Care Medicine, University Medical Center Hamburg-Eppendorf, Hamburg, Germany; 3 Research Center Borstel, Leibniz Lung Center, Borstel, Germany; Nitte University, INDIA

## Abstract

**Objectives:**

To determine the influence of local spread of clonal strains and testing of follow-up isolates on categorical (CA) and essential agreement rates (EA) of common colistin (COL) drug susceptibility testing methods with the broth microdilution (BMD) reference method.

**Methods:**

COL MICs were determined for 178 bacterial isolates (*Enterobacteriaceae*, n = 97; *Pseudomonas aeruginosa*, n = 81) collected within one year from 64 patients by BMD according to ISO standard 20776–1 (reference method), the SensiTest BMD panel (ST), agar dilution (AD), the VITEK 2 instrument, and gradient diffusion (GD) using antibiotic strips of two and Muller-Hinton agar plates of three manufacturers. CA and EA with BMD were calculated for all isolates and compared to the subset of 68 unique isolates.

**Results:**

CA ranges were 79.4% to 94.1% for the unique isolateq panel and 89.9% to 96.1% for all tested isolates. EA ranges were 64.7% to 86.8% and 67.4% to 91.0%, respectively. In both panels, EA for all GD assays was lower than 90%. Both lower and higher EA values ranging from—18.3% (MTS on BD agar) to + 6.3% (AD, Vitek 2) were observed in the full one-year sample. Acquisition of colistin resistance under therapy was observed for 3 patients.

**Conclusions:**

i) Repeat testing and local spread of clonal strains can positively or negatively affect CA and EA, ii) CA is more robust towards local influences than EA, iii) EA of GD and AD methods for COL with the reference BMD method is insufficient.

## Introduction

With the emergence of infections caused by multidrug-resistant Gram-negative bacteria (MDR-GNB), colistin (COL) has received significant attention as a last resort antimicrobial [1]. As administration of COL is associated with renal and, to a lesser extent, neurological toxicity, reliable antimicrobial susceptibility testing (AST) is crucial [2]. Recently, the European Committee for Antimicrobial Susceptibility Testing (EUCAST), issued a warning stating that i) gradient diffusion (GD) tests underestimate COL minimal inhibitory concentrations (MICs), ii) disk diffusion shall not be used for susceptibility testing, and iii) broth microdilution (BMD) is currently the only valid AST method [3].

In 2015, plasmid-encoded resistance to COL due to the phosphoethanolamine transferase MCR-1 has been reported for the first time in samples from livestock, food and humans [4]. Since then, the prevalence rates for MCR-1 positive isolates have been reported to range from only sporadic observations to 67% [5]. Although sufficient to confer resistance to COL in an *in vivo* infection model, MICs of isolates encoding MCR-1 (2–8 mg / L) are close to the current EUCAST resistance breakpoint (R > 2 mg / L), further emphasizing the relevance of reliable AST methods for correct isolate categorization [4]. Since the first description of transferable colistin resistance due to MCR-1, additional *mcr* genes have been described [6].

Most studies reporting performance data of COL AST methods are based on single patient isolates [7–9]. However, patients receiving COL are often chronically ill and thus likely to be sampled and tested repeatedly. In addition, clonal spread of causative species such as carbapenem-resistant *Pseudomonas aeruginosa* or *Klebsiella pneumoniae* within healthcare centers is well documented [10–14]. We wondered if categorial (CA) and essential agreement rates (EA) of COL AST methods, such as GD, agar dilution (AD), the SensiTest commercial BMD panel (ST) and the semiautomated Vitek 2 platform, with the reference standard (manual BMD according to ISO standard 20776–1, Table 1) were different in a “real-life” sample set, i.e. in all carbapenem-resistant MDR-GNB subjected to COL AST within one year including follow-up and clonal isolates, as compared to an “ideal” panel of unique bacterial isolates.

**Table 1:** Colistin AST methods compared in this study. VITEK 2 cards were incubated and evaluated automatically by the device, all other tests were incubated at 36 ± 2°C for 16–20 hours in ambient air. McF, McFarland standard; CAMHB, cation-adjusted Muller-Hinton II broth; QC, quality control; MHA, Muller-Hinton agar; BMD, broth microdilution; FSCA, Field Safety Corrective Action.

Test	Description	MIC range (mg / L)	Inoculum	Medium used in this study	Comment	Reference
Broth microdilution	In-house prepared serial dilution of colistin sulfate in untreated polystyrene 96-well plates	≤0.25 –>128	5 × 10^5^ CFU / mL	CAMHB (Sigma-Aldrich)	EUCAST and CLSI reference method; comparably labor intensive; single isolate test runs possible;	ISO 20776–1[15]
Agar dilution	In-house prepared serial dilution of colistin sulfate in untreated polystyrene 96-well plates	≤0.5 –>16	1 × 10^4^ CFU / spot	MHA (CAMHB + 1.7% Agar)	Comparably labor intensive; single isolate test runs possible by using 8-well microtiter strips;	Wiegand et al. [16]
Etest, MIC Test Strip	Agar gradient diffusion	≤0.016 –>256	McF 0.5 streaked with sterile swab	MHE (bioMérieux), MHA (Oxoid), MHA (BD)	Suitable for routine application; single isolate test runs possible; For Etest, use of MHE plates is mandatory; For Etest, only *Enterobacteriaceae* must be tested for diagnostic use; Comparably expensive;	FSCA 3061, FSCA 3206
VITEK 2	Card-based, semiautomated AST (manual inoculum preparation, automated incubation and reading)	≤0.5 –>16	McF 0.5–0.63	Contained in the test card	Suitable for routine application; single isolate test runs possible; Colistin susceptible test results require confirmation;	Dafopoulou et al.[17] FSCA 3490
SensiTest	Commercially available 4-test BMD panel containing dried colistin in 7 two-fold dilutions	≤0.25 –>16	5 × 10^5^ CFU / mL	CAMHB (Liofilchem, provided with assay)	Suitable for routine application; Comparably expensive; simultaneous AST of four isolates per panel required for optimum cost-efficiency;	Matuschek et al.[18]

## Methods

### Isolate collection, species identification, strain typing and inoculum preparation

All bacterial isolates were cultured from routine samples submitted to the bacteriology service at University Medical Center Hamburg-Eppendorf between September 1, 2015 and August 31, 2016. Species identification was performed on a Biotyper MALDI-TOF mass spectrometry system (Bruker, Bremen, Germany) and pure stock cultures were stored at -80°C. Clonal identity of bacterial isolates was assessed by pulsed-field gel electrophoresis (PFGE) as described previously [14,19,20]. For AST, stock cultures were thawed and passaged twice on MH agar plates. Inocula were prepared freshly in saline and adjusted to a 0.50 ± 0.05 McFarland (McF) turbidity standard using a calibrated Densichek device and polystyrene tubes (bioMérieux, Marcy-l'Étoile, France). *E*. *coli* ATCC 25922 (*mcr-1* negative), *E*. *coli* NCTC 13846 (*mcr-1* positive) and *P*. *aeruginosa* ATCC 27853 were used as controls for all AST assays.

### Broth microdilution

In-house BMD was performed according to ISO standard 20776–1 in untreated 96-well polystyrene plates (Greiner bio-one, Frickenhausen, Germany) using cation-adjusted Mueller Hinton II broth (CAMHB, Sigma-Aldrich, Munich, Germany) [15]. No additives were included in any part of the testing process (in particular, no polysorbate-80 or other surfactants). COL sulfate was obtained from Sigma-Aldrich (lot no. SLBQ0243V). The final inoculum was adjusted to 2–8 × 10^5^ CFU/ml. Correct inoculum densities were confirmed by obtaining CFU counts of appropriate inoculum dilutions on MH agar plates.

### Agar dilution

Agar powder (BactoAgar, BD) was added to CAMHB at a concentration of 17 g/L (1.7% agar) [16]. After autoclaving, the medium was aliquoted, cooled to 50°C and COL sulfate (Sigma-Aldrich) was added at appropriate concentrations to generate working solutions corresponding to a two-fold serial dilution. 100 μl of each aliquot were poured into the appropriate wells of untreated polystyrene 96-well plates. Plates were covered with sterile plastic lids, dried and stored in plastic bags in inverted position at 4 °C. The final inoculum was adjusted to 1 × 10^4^ CFU / well.

### Gradient diffusion

Inoculum suspensions were streaked on MHE agar (bioMérieux), MH agar (Oxoid, Wesel, Germany) and MH agar (Becton Dickinson, Heidelberg, Germany) using sterile cotton swabs. Gradient diffusion (GD) strips (Etest, bioMérieux, and MIC Test Strip, MTS, Liofilchem, Roseto degli Abruzzi, Italy) were placed on inoculated agar plates using a flame-sterilized forceps. Plates were incubated at 36 ± 2°C for 16–20 hours at ambient air. MIC endpoints were read according to manufacturer recommendations. MIC values between two-fold dilutions were rounded to the next two-fold dilution to allow comparison with the other AST assays.

### VITEK 2

AST on the Vitek 2 system (bioMérieux) was performed using AST-N248 cards (lot no. 6480147103). Inocula (McF 0.50 ± 0.05 in 0.45% saline) and AST cards were loaded in the device for incubation and MIC values were determined automatically. MIC values were manually extracted from the device software for further analysis.

### SensiTest

SensiTest COL panels (Liofilchem) were inoculated according to manufacturer recommendations using CAMHB supplied with the test panels. Panels were sealed and incubated at 36 ± 2°C for 16–20 hours in ambient air.

### Detection of *mcr* genes

DNA was extracted from pure bacterial cultures on the Qiasymphony SP (Qiagen, Hilden, Germany) instrument using QIAsymphony mericon bacteria chemistry. For detection of *mcr-1*, quantitative-realtime PCR was performed according to the protocol of Chabou et al. [21] using Quantifast pathogen + IC kit chemistry (Qiagen) on a Lightcycler 480 II instrument (Roche, Mannheim, Germany). For detection of *mcr-2*, *-3*, *-4*, and *-5* a quantitative-realtime PCR was designed using the BeaconDesigner software (PRIMIER Biosoft, Palo Alto, USA) and consensus sequences available at the NCBI nucleotide database. Amplification of the 23S rRNA gene using primersTACYCYGGGGATAACAGG and TACYCYGGGGATAACAGG, and probe FAM-TTGGCACCTCGATGTCGG-BHQ1 was performed as extraction control [22]. DNA extracts from *E*. *coli* ATCC 25922 (*mcr-1* negative), *E*. *coli* NCTC 13846 (*mcr-1* positive), *E*. *coli* KP37 (*mcr-2* positive), *E*. *coli* 2013-SQ352 (*mcr-3* positive), *E*. *coli* DH5α with the entire *mcr-4* gene cloned in the pCR2 vector and *Salmonella Paratyphi* B 13-SA01718 (*mcr-5* positive) were used as positive controls [6].

### Data analysis

MICs were interpreted according to the EUCAST breakpoint table, version 7.1 (MIC ≤ 2 mg / L, susceptible; MIC > 2 mg / L, resistant). Categorical agreement (CA) was defined as the percentage of isolates with identical MIC interpretation in BMD and the comparator method. Essential agreement (EA) was defined as the percentage of isolates with MICs within 1 doubling dilution from the reference method MIC. To allow comparability, calculation of EA was performed after reinterpretation of all MIC values according to the Vitek 2 and AD MIC range (≤ 0.5 –> 16 mg / L, the narrowest MIC range of all assays).

## Results

A total of 178 carbapenem-resistant MDR-GNB from 64 patients were included in the study. The mean number of follow-up samples tested per patient was 1.2 (range 0 to 13). In addition, nosocomial transmission was observed for two carbapenemase producing strains (Oxa-48 and CTX-M 14 producing *K*. *pneumoniae*, VIM-2 producing *P*. *aeruginosa*) during the study period, resulting in 68 unique patient isolates (4 patients were tested positive for 2 strains) [14]. In three patients infected with *K*. *pneumoniae* (n = 2) or *P*. *aeruginosa* (n = 1), development of resistance to COL was observed during therapy. None of the tested isolates was positive for the investigated *mcr* alleles. Main characteristics of the whole one-year isolate panel and the subset of unique isolates are summarized in Table 2.

**Table 2:** Characteristics of the isolates used in this study. Susceptibility and resistance to colistin is reported as observed in the reference in-house broth microdilution method.

Species	full one-year sample					unique isolate subset				
	n			Median MIC (mg / L)	MIC range (mg / L)	n			median MIC (mg / L)	MIC range (mg / L)
	total	Susceptible (%)	Resistant (%)			total	Susceptible (%)	Resistant (%)		
*Enterobacteriaceae*	97	66 (68.0)	31 (32.0)	0.5	0.5–32	24	21 (87.5)	3 (12.5)	0.5	0.5–16
*P*. *aeruginosa*	81	76 (93.8)	5 (6.2)	0.5	0.5–32	44	41 (93.2)	3 (6.8)	0.5	0.5–32
total	178	142 (79.8)	36 (20.2)	0.5	0.5–32	68	62 (91.2)	6 (8.8)	0.5	0.5–32

For the investigated *Enterobacteriaceae*, CA ranged between 87.5% (MTS on all tested MH media) and 95.8% (ST) in the unique isolate panel (n = 24), and between 89.7% (MTS on Oxoid MH) and 99.0% (ST) in all tested isolates (n = 97, Table 3). For *P*. *aeruginosa*, CA ranges were 72.7% (Etest on Oxoid MH) to 97.7% (MTS on MHE and BD MH) for the unique isolate panel (n = 44) and between 80.2% (Etest on Oxoid MH) and 97.5% (MTS on MHE and BD MH) in all tested isolates (n = 81). Overall, CA ranges were 79.4% (Etest on Oxoid MH) to 94.1% (Vitek 2, MTS on MHE and BD MH) for all unique isolates (n = 68) and 89.9% (Etest on Oxoid MH) to 96.1% (Vitek 2, Etest on MHE) for all tested isolates (n = 178).

**Table 3:** Percent categorical agreement (CA) between comparator methods for colistin MIC determination and the reference broth microdilution method.

	n	AD	Etest / MHE	Etest / Oxoid	Etest / BD	MTS / MHE	MTS / Oxoid	MTS / BD	Vitek2	ST
*Enterobactericeae*^1^	24	91.7%	91.7%	91.7%	91.7%	87.5%	87.5%	87.5%	91.7%	95.8%
*Enterobactericeae*^2^	97	95.9%	96.9%	97.9%	95.9%	92.8%	89.7%	90.7%	96.9%	99.0%
*P*. *aeruginosa*^1^	44	86.4%	93.2%	72.7%	77.3%	97.7%	93.2%	97.7%	95.5%	88.6%
*P*. *aeruginosa*^2^	81	86.4%	95.1%	80.2%	85.2%	97.5%	95.1%	97.5%	95.1%	90.1%
Total^1^	68	88.2%	92.6%	79.4%	82.4%	94.1%	91.2%	94.1%	94.1%	91.2%
Total^2^	178	91.6%	96.1%	89.9%	91.0%	94.9%	92.1%	93.8%	96.1%	94.9%

^1^ unique isolate subset

^2^ full one-year sample

EA ranged between 79.2% (MTS on Oxoid and BD MH) and 87.5% (AD, Etest on all MH, Vitek 2, ST) in all unique *Enterobacteriaceae* and between 60.8% (MTS on BD MH) and 94.8% (ST) in the full isolate panel (Table 4). For *P*. *aeruginosa*, EA ranges were 52.3% (AD) to 88.6% (MTS / MHE), and 40.7% (AD) to 86.4% (ST), respectively. Overall, EA ranged between 64.7% (AD) and 86.8% (MTS / MHE) for all unique isolates and between 67.4% (MTS / Oxoid) and 91.0% (ST) for all isolates tested during the study period.

**Table 4:** Percent essential agreement (EA) between comparator methods for colistin MIC determination and the reference broth microdilution method.

	n	AD	Etest / MHE	Etest / Oxoid	Etest / BD	MTS / MHE	MTS / Oxoid	MTS / BD	Vitek 2	ST
*Enterobactericeae*^1^	24	87.5%	87.5%	87.5%	87.5%	83.3%	79.2%	79.2%	87.5%	87.5%
*Enterobactericeae*^2^	97	93.8%	88.7%	87.6%	85.6%	64.9%	62.9%	60.8%	93.8%	94.8%
*P*. *aeruginosa*^1^	44	52.3%	72.7%	54.5%	61.4%	88.6%	72.7%	84.1%	84.1%	84.1%
*P*. *aeruginosa*^2^	81	40.7%	74.1%	49.4%	61.7%	85.2%	72.8%	80.2%	85.2%	86.4%
Total^1^	68	64.7%	77.9%	66.2%	70.6%	86.8%	75.0%	82.4%	85.3%	85.3%
Total^2^	178	69.7%	82.0%	70.2%	74.7%	74.2%	67.4%	69.7%	89.9%	91.0%

^1^ unique isolate subset

^2^ full one-year sample

Calculations based on the full isolate panel mostly resulted in higher CA as compared to the unique isolate panel (range + 0.1% to + 10.5%) with the exception of *P*. *aeruginosa* and MTS / MHE, MTS / BD and Vitek 2 where CA was slightly lower (- 0.2%, - 0.2% and—0.4%, respectively). For EA, deviations towards both higher and lower values were observed in the full as compared to the unique sample set ranging from—18.3% (MTS / BD) to + 6.3% (AD, Vitek 2).

## Discussion

The purpose of this study was to investigate the impact of follow-up testing and spread of single bacterial clones on CA and EA of COL AST as compared to the ISO standard 20776–1 reference BMD method. With respect to CA, most assays performed acceptably based on both the full one-year sample and the subset of unique isolates with CA rates close to or above 90%. One exception was Etest with Oxoid or BD MH media in *P*. *aeruginosa*, supporting the recommendation by the manufacturer to use MHE medium for this GD assay only. Of note, classification errors in *P*. *aeruginosa* may in part also relate to the fact that the breakpoint indicating susceptibility (MIC ≤ 2 mg / L) splits the wildtype population (epidemiological cut-off = 4 mg / L) [9]. With respect to EA, test performance showed significant variation. Firstly, our study corroborates previous observations that COL MICs from AD testing show insufficient agreement with MICs derived from BMD, especially for *P*. *aeruginosa* [17]. Secondly, our findings regarding GD assays are in accordance with a recent study by Matuschek et al. who studied a panel of 75 unique clinical isolates and also found low EA rates (43 to 71%) depending on GD and Mueller-Hinton agar manufacturers [9]. In our hands, EA values for Etest were lower in *P*. *aeruginosa* (irrespective of the medium) whereas EA values for MTS were lower in the tested *Enterobacteriaceae*. Interestingly, when results from the full one-year sample are compared to those of the unique isolate subset, EA values were found to be lower for MTS on all media while EA values were higher for other assays such as Etest, Vitek 2, and ST. This finding demonstrates that local factors can increase or decrease EA rates. In contrast, CA appears to be more robust with respect to local influences.

This study has some important limitations. Firstly, strain clonality was investigated using PFGE. While PFGE is a well-established method for outbreak investigations, current efforts are ongoing to allow more detailed investigations of clonal relationships between strains using whole genome sequencing. Secondly, the reported impact of outbreak events and testing of follow-up samples on EA and CA is specific to our local epidemiology and re-testing policy and cannot be generalized. However, our data clearly demonstrate that laboratories performing AST of COL should be aware that generic performance parameters derived from collections of “non-duplicate” bacterial isolates may not be directly applicable to their routine conditions.

In conclusion, our data show that testing a complete one-year sample of carbapenem-resistant MDR-GN bacteria did not negatively influence CA for the investigated comparator methods, e.g. by overrepresentation of discrepant isolates. The impact on EA, which by definition is more sensitive towards minor MIC changes than CA, is more difficult to predict and both increased and decreased agreement rates must be expected.

## Supporting information

S1 Table Phenotypic drug susceptibility testing results for control strains used in this study. All MIC values in mg/L.
(XLSX)

S2 Table PCR results for /mcr/-1, /mcr/-2, /mcr/-3, /mcr/-4 and /mcr/-5. PCRs were performed for one isolate per patient.
(XLSX)
